# Myositis: A Rare First Presentation of Systemic Lupus Erythematosus

**DOI:** 10.7759/cureus.70090

**Published:** 2024-09-24

**Authors:** Mariam K Ibrahim, Abdullah S Mousa, Ali M Al-Azzawi

**Affiliations:** 1 Rheumatology, Samarra General Hospital, Samarra, IRQ; 2 Rheumatology, Al Karkh General Hospital, Baghdad, IRQ; 3 Nephrology, Al-Karama General Hospital, Baghdad, IRQ

**Keywords:** rheumatology, muscle weakness, vasculitis, myositis, sle, systemic lupus erythematosus

## Abstract

Systemic lupus erythematosus (SLE) is a chronic autoimmune disease that affects multiple systems with a variety of clinical manifestations and serological abnormalities. Overt myositis is a rare and not well-studied manifestation of SLE, which is associated with a more severe disease course and may be overlooked by clinicians. This case report describes a rare first presentation of SLE with myositis.

An 18-year-old female patient presented with a three-month history of generalized muscle weakness, polyarthralgia, and rashes. Physical examination revealed malar rash, a dry scaly pigmented rash affecting the flanks, and a non-blanching purpuric rash with mottled discoloration and a well-defined ulceration, affecting both hands, suggestive of vasculitis. A pigmented atrophic patch on the right upper chest was also suggestive of discoid lupus. Further examination findings included bilateral upper and lower limb weakness affecting proximal muscles (Medical Research Council (MRC) grade 3/5) more than the distal muscles (MRC grade 4/5).

The patient's investigation panel revealed leukopenia, anemia, elevated liver enzymes, and elevated erythrocyte sedimentation rate (ESR) and C-reactive protein (CRP) as well as significant elevation in creatinine kinase. Further antibody testing revealed positive antinuclear antibodies (ANA), anti-double-stranded DNA (anti-dsDNA), anti-Smith, and anti-U1-ribonucleoprotein (U1RNP), along with electrodiagnostic study supporting the diagnosis of SLE complicated by myositis and vasculitis.

Treatment was initiated with prednisolone, hydroxychloroquine, methotrexate, folic acid, and omeprazole with sunscreen. Over the next several months, the patient demonstrated significant clinical and laboratory improvement, regaining full muscle power, with her vasculitis rash also improving and steroid tapering initiated to avoid side effects.

This case highlights the importance of recognizing myositis as a rare potential first presentation of SLE and the need for heightened clinical awareness as early diagnosis and treatment is vital for improving long-term outcomes. This case adds to the existing literature and provides a reference for future clinical encounters with such complex cases.

## Introduction

Systemic lupus erythematosus (SLE) is a chronic autoimmune disease that affects multiple systems with a variety of both clinical manifestations and serological abnormalities and an array of antibody production [1]. Throughout the clinical course of the disease, which is highly variable, it is frequently encountered by unpredictable flares. The condition can present from mild joint disease to more serious hematological, renal, or nervous system involvement, making the condition challenging to diagnose and manage [1,2].

As myositis is not a common presentation of SLE, it tends to be missed or overlooked [3]. Myositis has been suggested to be linked with more severe disease, and a prevalence of muscle disease ranging from 3% to 8% is reported in adult patients [4]. This case report studies a rare presentation of SLE in an 18-year-old female patient with myositis and vasculitis.

## Case presentation

An 18-year-old female patient presented to the rheumatology outpatient clinic with generalized weakness along with a history of fatigue, polyarthralgia, and rash affecting the face, hands, trunk, and flanks for three months. The patient reported no shortness of breath or difficulty swallowing.

**Figure 1:**
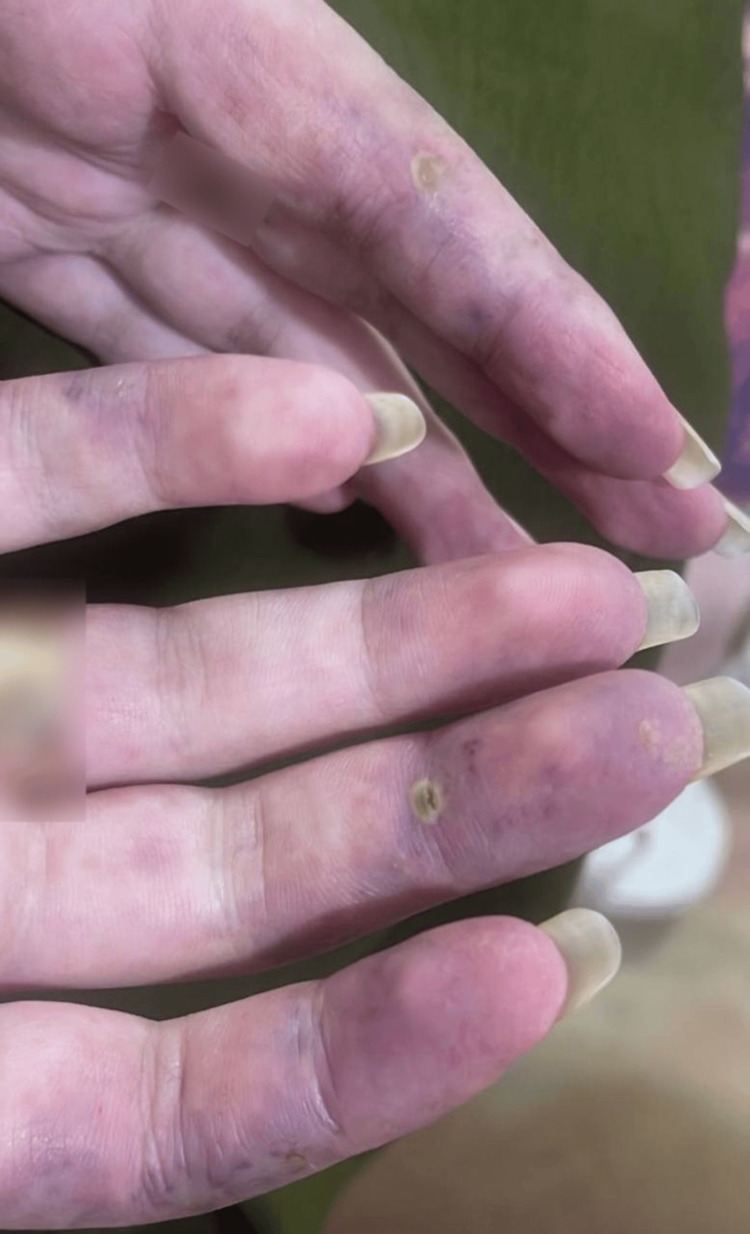
Rash and ulceration affecting the hands Non-blanching purpuric rash with mottled discoloration and a well-defined ulceration with surrounding erythema, measuring 1 cm, affecting both hands, highly suggestive of vasculitis

Physical examination revealed a malar rash over the face and eyelids. The patient also had bilateral upper and lower limb weakness affecting proximal muscles (Medical Research Council (MRC) grade 3/5) more than distal muscles (MRC grade 4/5). Further assessment revealed a non-blanching purpuric rash with mottled discoloration and a well-defined ulceration with surrounding erythema, measuring 1 cm, affecting both hands as seen in Figure 1. A scaly, dry, pigmented rash was also noted on the flanks. Additionally, a hyperpigmented atrophic patch on the right upper chest was suggestive of discoid lupus.

A full panel of investigations was ordered, and Table 1 presents the results.

**Table 1:** Baseline investigation panel CBC = complete blood count, WBC = white blood cells, HB = hemoglobin, LFT = liver function test, RFT = renal function test, GOT = glutamate oxaloacetate transaminase, GPT = glutamate pyruvate transaminase, ESR = erythrocyte sedimentation rate, CRP = C-reactive protein, CPK = creatine phosphokinase, ANA = antinuclear antibodies, anti-dsDNA = anti-double-stranded DNA, U1RNP = U1-ribonucleoprotein

Category	Parameter	Result	Reference range
CBC	WBC	3.2×10^9^/L	4-11×10^9^/L
	Lymphocytes	0.5×10^9^/L	1-3×10^9^/L
	HB	8.3 g/dL	13.5-17.5 g/dL (males)
	Platelets	181×10^9^/L	150-450×10^9^/L
LFT	GOT	306.6 U/L	<40 U/L
	GPT	91.3 U/L	<40 U/L
RFT	Urea	3.76 mmol/L	2.8-7.2 mmol/L
	GUE	No protein, no cast	Normal
Inflammatory marker	ESR	55 mm/hour	<20 mm/hour
	CRP	14.5 mg/L	<5 mg/L
	CPK	1,295 U/L	0-150 U/L
Antibody profile	ANA	+ve	
	Anti-dsDNA	+ve	
	Anti-Smith	+ve	
	U1-RNP	+ve	
	Ku	+ve	
	Anti-histone	+ve	
Blood film	Bicytopenia, normochromic anemia, leukopenia		
Antiphospholipid screen	Negative		

X-ray of the hands revealed no calcinosis present. Electromyography and nerve conduction studies revealed normal insertional activity, spontaneous activity in the form of FIBS (fibrillation potentials) and positive sharp waves (PSW) grade 2-3 in all selected muscles, and myopathic motor unit action potential (MUAP) (small amplitude, short duration polyphasic, and early recruitment) in proximal muscles mainly. An electrodiagnostic study showed evidence of diffuse chronic symmetric myopathic process affecting both upper and lower limbs, and the intermediate and proximal muscles are maximally involved with signs of active membrane instability. The study findings along with the clinical history, examination, and laboratory values support the diagnosis of inflammatory myopathy.

The patient was diagnosed with systemic lupus erythematosus (SLE) as her clinical manifestations, laboratory values, and immunological profile fulfill the 2019 European League Against Rheumatism (EULAR)/American College of Rheumatology (ACR) Classification Criteria for Systemic Lupus Erythematosus [1].

Treatment was initiated and directed toward managing SLE and myositis, as well as managing complications. The patient was started on the following regimen with dermatological department support: daily prednisolone 40 mg and hydroxychloroquine 200 mg, oral methotrexate 15 mg weekly, and folic acid 5 mg three times a week with gastroprotection in the form of daily omeprazole 40 mg. The patient was also instructed to use +50 SPF sunscreen. Additionally, a baseline DEXA scan was arranged, and bone protection was initiated in the form of a weekly alendronate tablet 70 mg along with calcium and vitamin D supplementation. The patient was advised to avoid live vaccines and referred for influenza and pneumococcal vaccination.

The patient was followed up regularly at the rheumatology outpatient clinic for monitoring, checking compliance, and managing any new symptoms or complications with laboratory monitoring. Over the following two weeks, the patient's facial rash had started to improve, as well as the rash and ulcers of the hands. Further follow-up revealed improvement in muscle power (MRC grade 4/5). The patient was advised to continue the current treatment.

Over the next two months, the patient's improvement continued as her muscle power returned to normal (MRC grade 5/5), and the ulcers on the hands also completely resolved with some residual hyperpigmentation. A steroid taper was, therefore, initiated to avoid steroid-induced side effects. The following month, the patient displayed more improvement as her laboratory investigations displayed significant improvement over baseline as seen in Table 2.

**Table 2:** Investigation panel after treatment showing marked laboratory improvement over baseline WBC = white blood cells, HB = hemoglobin, ESR = erythrocyte sedimentation rate, AST = aspartate aminotransferase, ALT = alanine aminotransferase, CRP = C-reactive protein, CPK = creatine phosphokinase

Investigation	Result	Reference range
WBC	9.7×10^9^/L	4-11×10^9^/L
HB	11.7 g/dL	12-16 g/dL
Platelets	329×10^9^/L	150-450×10^9^/L
ESR	18 mm/hour	<20 mm/hour
Urea	28 mg/dL	7-20 mg/dL
Creatinine	0.5 mg/dL	0.6-1.2 mg/dL
AST	24.4 U/L	10-40 U/L
ALT	27.6 U/L	7-56 U/L
CRP	3 mg/L	<5 mg/L
CPK	105 U/L	20-200 U/L

**Figure 2:**
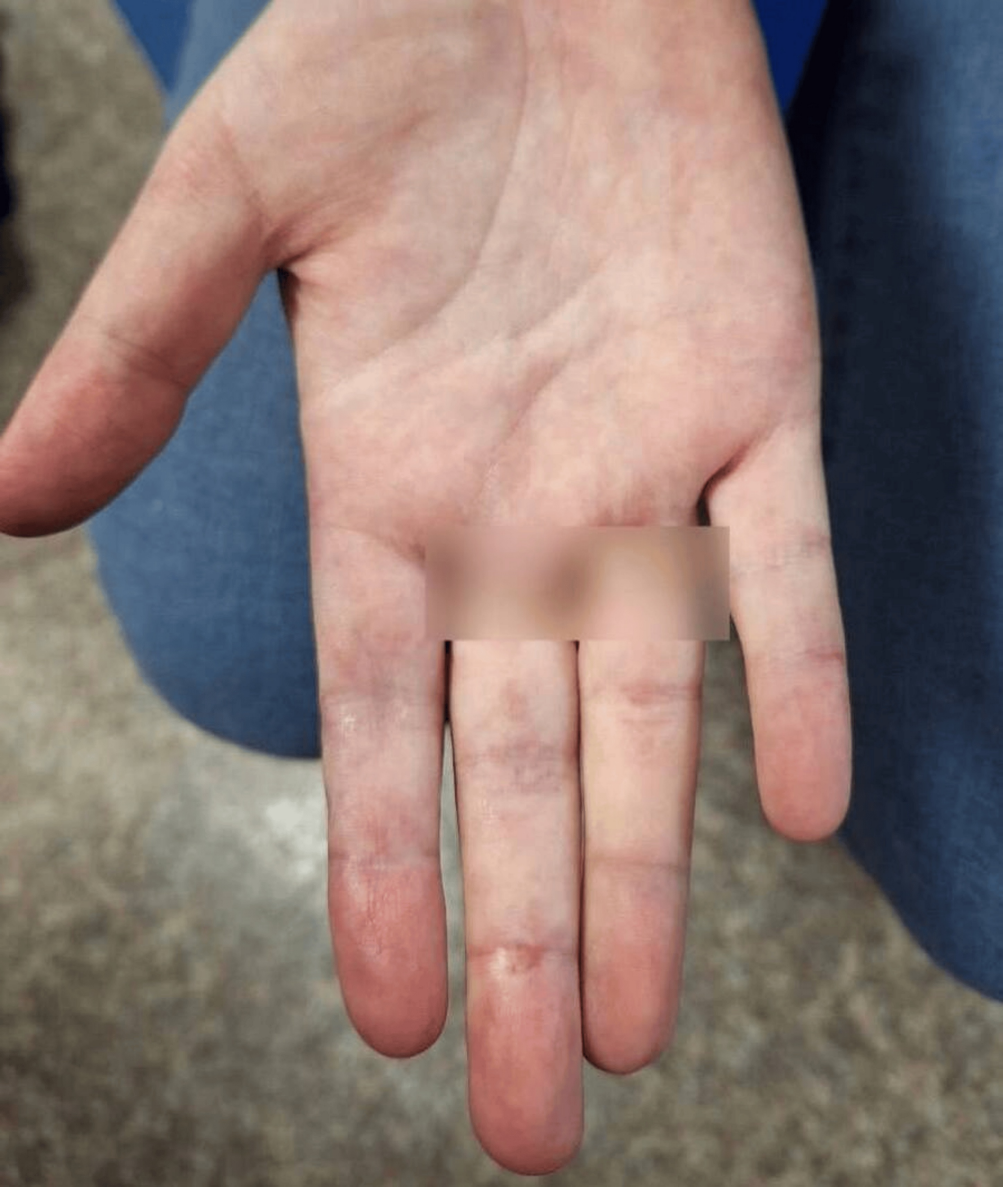
Marked improvement of rash and ulceration after treatment Improvement of the rash with resolved mottled discoloration and ulceration. The patient's skin appears more evenly colored, with some residual hyperpigmentation and no signs of purpura, indicating healing and resolution of the vasculitis of the hands following treatment.

The patient was advised to continue steroid tapering while continuing her other medications with further follow-up visits scheduled. Figure 2 highlights the marked improvement of her rash with resolved mottled discoloration and ulceration. The patient's skin appears more evenly colored, with some residual hyperpigmentation and no signs of purpura, indicating healing and resolution of the vasculitis of the hands following treatment.

## Discussion

This case represents a complex and rare presentation of SLE in an 18-year-old female patient with myositis. Muscle involvement in SLE is common and occurs in the form of myalgia, tenderness, or weakness and can be a reason for seeking medical attention [3]. Overt myositis with significant elevation in creatinine kinase (≥500 unit/L) is rare and is reported to occur in less than 5% of patients [5]. The presentation with myositis in this SLE case makes it notable as it was the muscle weakness and difficulty with daily tasks such as standing up from a chair and combing the hair that prompted the patient to seek medical attention. The other symptoms, including the rash, fatigue, and arthralgia, were neglected by the patient.

The patient's clinical manifestations, including malar rash, discoid lupus, and vasculitis rash, along with laboratory findings showing anemia and leukopenia, as well as immunological profile positive for ANA, anti-dsDNA, and anti-Smith antibodies, both highly specific for SLE, support the diagnosis of systemic lupus erythematosus, therefore fulfilling the 2019 EULAR/ACR classification criteria for SLE [1].

Diagnosing myositis in SLE can be challenging as there are many causes for muscle weakness, including medications used for SLE treatment such as glucocorticoids and anti-malarial drugs. However, these are not associated with significant elevations in creatinine kinase [5]. A thorough clinical history, physical examination, and laboratory investigations coupled with electromyography and nerve conduction studies are necessary to identify SLE-induced myositis. This patient presented with proximal muscle weakness, significant elevation in CK levels (1,295 unit/L), elevated liver enzymes, and elevated inflammatory markers with electrodiagnostic studies revealing diffuse chronic myopathic process. These findings, along with her immunological profile, support the diagnosis of inflammatory myopathy.

The development of vasculitis in SLE can take the form of cutaneous vasculitis. Small vessels are frequently affected in most cases. Medium vessel vasculitis occurs less and presents as subcutaneous nodules or ischemic ulcers. Literature shows that myositis with hematological abnormalities such as anemia, Coombs' positivity, leucopenia, anti-Smith, and anti-ribonucleoprotein (RNP) may predict the development of cutaneous vasculitis as seen in this case [6]. Literature also suggests a possible link between anti-Smith antibodies and the development of myositis [3].

Management of such complex cases requires aggressive treatment to be started early to induce remission and prevent complications as the presence of myositis has been reported to be associated with poor SLE outcomes [3]. The treatment involved medium- to high-dose glucocorticoids, hydroxychloroquine, methotrexate, and folic acid, with dermatological support. The patient response was closely monitored, and adjustments were made to taper steroids to avoid steroid-induced side effects [7]. This approach has led to significant improvement in the patient's clinical symptoms and laboratory values.

This case aims to highlight the importance of recognizing myositis as a possible presentation of SLE as this is not a well-recognized presentation. It may be overlooked by clinicians, which can delay treatment [3]. Early diagnosis and aggressive management of SLE-related myositis and vasculitis are vital to prevent irreversible damage and further deterioration and improve long-term outcomes [1].

## Conclusions

This case report highlights a rare presentation of SLE with a predominance of myositis. The diagnosis can be challenging as overt myositis is an uncommon manifestation of SLE, which may be overlooked by clinicians, emphasizing the importance of performing thorough clinical evaluation and comprehensive laboratory testing. Successful management of this patient was mainly accredited to the early diagnosis, which enabled prompt initiation of aggressive treatment.

This case report presents full details about the diagnostic approach, course of the disease, treatment strategies, and follow-up after successful management, reinforcing the needed awareness about the atypical presentation of SLE and individualized patient care for optimized outcomes. Additionally, it contributes to the existing medical literature regarding this rare manifestation and provides a reference for future clinical practice in such complex cases.
